# Intradermal influenza vaccination of healthy adults using a new microinjection system: a 3-year randomised controlled safety and immunogenicity trial

**DOI:** 10.1186/1741-7015-7-13

**Published:** 2009-04-02

**Authors:** Jiri Beran, Arvydas Ambrozaitis, Alvydas Laiskonis, Narseta Mickuviene, Patrick Bacart, Yvan Calozet, Etienne Demanet, Stephane Heijmans, Paul Van Belle, Françoise Weber, Camille Salamand

**Affiliations:** 1 Vaccination and Travel Medicine Centre, Hradec Kralove, Czech Republic; 2 Department of Infectious Diseases, Dermatovenerology and Microbiology, Vilnius University, Vilnius, Lithuania; 3 Kaunas University of Medicine, Kaunas 2 Clinical Hospital, Kaunas, Lithuania; 4 Institute of Psychophysiology and Rehabilitation, Kaunas University of Medicine, Lithuania; 5 ResearchLink, a Clinical Trial Network of GPs, Thuin, Belgium; 6 General Practitioner, Private Practice, Molenbeek, Belgium; 7 General Practitioner, Private Practice, Gribomont, Belgium; 8 General Practitioner, Private Practice, Thuin, Belgium; 9 General Practitioner, Private Practice, Linkebeek, Belgium; 10 General Practitioner, Private Practice, Kraainem, Belgium; 11 Sanofi Pasteur, Lyon, France

## Abstract

**Background:**

Intradermal vaccination provides direct and potentially more efficient access to the immune system via specialised dendritic cells and draining lymphatic vessels. We investigated the immunogenicity and safety during 3 successive years of different dosages of a trivalent, inactivated, split-virion vaccine against seasonal influenza given intradermally using a microinjection system compared with an intramuscular control vaccine.

**Methods:**

In a randomised, partially blinded, controlled study, healthy volunteers (1150 aged 18 to 57 years at enrolment) received three annual vaccinations of intradermal or intramuscular vaccine. In Year 1, subjects were randomised to one of three groups: 3 μg or 6 μg haemagglutinin/strain/dose of inactivated influenza vaccine intradermally, or a licensed inactivated influenza vaccine intramuscularly containing 15 μg/strain/dose. In Year 2 subjects were randomised again to one of two groups: 9 μg/strain/dose intradermally or 15 μg intramuscularly. In Year 3 subjects were randomised a third time to one of two groups: 9 μg intradermally or 15 μg intramuscularly. Randomisation lists in Year 1 were stratified for site. Randomisation lists in Years 2 and 3 were stratified for site and by vaccine received in previous years to ensure the inclusion of a comparable number of subjects in a vaccine group at each centre each year. Immunogenicity was assessed 21 days after each vaccination. Safety was assessed throughout the study.

**Results:**

In Years 2 and 3, 9 μg intradermal was comparably immunogenic to 15 μg intramuscular for all strains, and both vaccines met European requirements for annual licensing of influenza vaccines. The 3 μg and 6 μg intradermal formulations were less immunogenic than intramuscular 15 μg. Safety of the intradermal and intramuscular vaccinations was comparable in each year of the study. Injection site erythema and swelling was more common with the intradermal route.

**Conclusion:**

An influenza vaccine with 9 μg of haemagglutinin/strain given using an intradermal microinjection system showed comparable immunogenic and safety profiles to a licensed intramuscular vaccine, and presents a promising alternative to intramuscular vaccination for influenza for adults younger than 60 years.

**Trial registration:**

Clinicaltrials.gov NCT00703651.

## Background

Annual influenza epidemics cause substantial morbidity and mortality in all segments of the population, although some groups, such as elderly adults and individuals of any age with certain chronic conditions are especially at risk [1,2]. Accordingly, international and national guidelines recommend influenza vaccination primarily for these groups, as well as for groups such as young children, pregnant women, healthcare workers and any person living with someone at risk [3,4].

In healthy adults younger than 60 or 65 years (the threshold age for the 'elderly' recommendation in most national recommendations), influenza vaccination coverage remains low despite being the most cost-effective intervention against annual influenza infection [5]. In a survey of influenza vaccination coverage and motivations in five European countries, the vaccination rate in individuals aged around 14 to 65 years was less than 15% [6]. The results of this survey also suggested that the availability of convenient alternative vaccination methods to standard intramuscular (IM) vaccination would encourage increased uptake. Intradermal (ID) vaccination might represent one such alternative method. Due to its ease of accessibility, recent research for an alternative way of administering inactivated influenza vaccine has focused on delivery into the skin, either into or through the epidermis (referred to as epidermal, transcutaneous or transdermal vaccination) or into the dermis (ID vaccination) [7,8]. The skin is not only a sensorial organ and a physical barrier between the body and its environment; it is also an efficient immunological barrier, screening invading molecules and particles to stimulate appropriate immunological responses. It is equipped with populations of professional antigen presenting cells, including Langerhans cells in the epidermis and dermal dendritic cells in the dermis, as well as a network of draining lymph vessels that start in the dermis [9,10]. Some of the mechanisms of the immune response to an ID injection remain to be elucidated and depend on multiple factors, including the nature of the antigen and the immune status of the host. It is thought that an ID injection results in two complementary mechanisms for the presentation of antigen and activation of T-cells in the lymph node: i) the capture and transport of antigen by dendritic cells in the dermis (predominantly dermal dendritic cells, although other cells populations may be recruited to the dermis) to the draining lymph nodes, and ii) the direct migration of free antigen through the lymph ducts to the nodes where it is captured by lymph node resident dendritic cells. It is also possible that some of the antigen migrates outwards to the epidermis where it would be captured by Langerhans cells [11]. ID vaccination delivers antigen directly to this immune system and has been shown to be effective for a range of vaccines, including rabies [12-14], hepatitis B [15-17] and influenza [18-21]. However, to date the use of this route has been hampered by the lack of appropriate vaccine delivery systems combining reliability, safety and simplicity of use. The standard technique to administer intradermal vaccines such as rabies is difficult to perform correctly and requires specifically trained and experienced personnel.

A new microinjection system (Soluvia™, BD, Becton, Dickinson and Company, Franklin Lakes, NJ, USA) has been developed specifically to provide a convenient and reliable ID vaccination that overcomes the technical difficulties associated with historical ID injection methods. This easy-to-use system features a prefilled, ready-to-use syringe with micro-needle that protrudes 1.5 mm from the proximal end of the glass syringe [22]. It has been shown to result in the consistent and accurate dermal infiltration of the intended volume of fluid [22].

We report a trial conducted to assess the immunogenicity and safety of a trivalent, inactivated, split-virion influenza vaccine given intradermally using the new microinjection system. Several ID dosages were evaluated in comparison with a licensed inactivated influenza vaccine given intramuscularly. The trial lasted 3 years to investigate the safety of annual revaccination with the ID vaccine and annual vaccination alternating between ID and IM routes.

## Methods

The study was conducted in five centres in Belgium, one in the Czech Republic (Years 1 and 2 only) and three centres in Lithuania between September 2003 and May 2006. It was approved by the ethics committee of each centre before any patients were enrolled at that centre, and was conducted in accordance with the Declaration of Helsinki and Good Clinical Practice. All subjects gave written informed consent before entering the study.

### Study subjects

Healthy volunteers aged 18 to 57 years (that is, <60 years at Year 3 of the study) were eligible. Exclusion criteria were: allergies to egg or chicken proteins or any of the vaccine constituents; a chronic illness in the active phase; acute febrile disease within 72 hours of any of the vaccinations or an axillary temperature >37.5°C on the day of vaccination; influenza vaccination within 6 months of the first study vaccination; any vaccination within 28 days of each study vaccination; pregnancy or breastfeeding; treatment with immunosuppressive or cancer therapy within 1 month of each vaccination, or an immunoglobulin injection within 3 months of each vaccination.

### Study design

This was a phase II, multicentre, randomised, partially blinded, dose-ranging study conducted over 3 successive years to assess the immunogenicity and safety of three annual vaccinations of ID, trivalent, inactivated, split-virion influenza vaccine in comparison with a licensed IM control vaccine. Each year, vaccination of all subjects occurred over a period of approximately 2 months (September and October). The primary objective of the study was to demonstrate, after vaccination in the first year of the study, that an ID vaccination of 3 μg or 6 μg of haemagglutinin (HA) per vaccine strain induced a non-inferior immune response compared with the IM control vaccine for all three vaccine strains. Secondary objectives included: assessment of compliance with the immunogenicity criteria defined in the European Medicines Evaluation Agency (EMEA) Note for Guidance [23], description of injection site and systemic safety after vaccination, and assessment on safety of the effect of three (Years 1, 2 and 3) annual ID vaccinations or alternating ID and IM vaccinations from year to year.

In Year 1, 1150 subjects were randomised equally into three groups and received a first injection of either 3 μg or 6 μg of HA/strain intradermally or the IM control vaccine (15 μg HA/strain). The initial plan was to select either the 3 μg or 6 μg dosage based on the immunogenicity results from this first year to proceed with in Years 2 and 3. Subjects were to be randomised again in Year 2 and a third time in Year 3 into two equal groups and receive either the chosen ID dosage or the IM control vaccine. Statistical analysis performed with immunogenicity data collected after Year 1 vaccination showed that neither the 3 μg nor the 6 μg ID vaccine met the criteria for non-inferiority to the control IM vaccine. The protocol was therefore amended to continue vaccination in Years 2 and 3 with an escalated ID dose of 9 μg of HA/strain. The rationale for this amendment was two-fold: firstly to obtain descriptive immunogenicity data in a large sample size with this 9 μg ID formation before performing a formal assessment of non-inferiority versus the IM control in a follow-up trial, and secondly to assess the effect on safety of three annual ID vaccinations or alternating ID and IM vaccinations from year to year, as initially planned. This report will concentrate on the 9 μg vaccine formulation.

**Figure 1:**
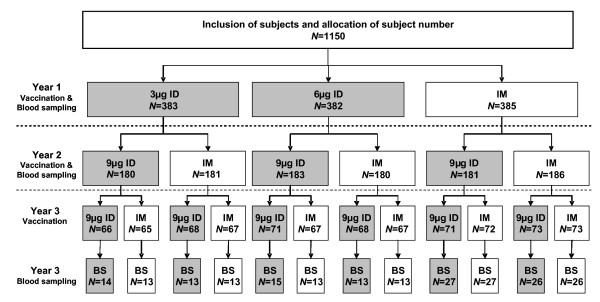
**Randomisation strategy**. Study subjects were randomised before each of the three vaccinations (represented by arrows): included subjects were randomised into three equally sized vaccine groups for the Year 1 vaccination, then in two equally sized vaccine groups in each of the subsequent years. Randomisation for vaccination in Years 2 and 3 was stratified for the vaccine received in previous years to ensure that a comparable number of subjects were included in each vaccine group. An additional Year 3 immunogenicity randomisation list was generated to select randomly a subset of approximately 30 subjects per vaccine stratum for blood sampling. Randomisation was also stratified for centre (not illustrated here). *N *= number of subjects randomised to each group (discontinuations are not represented).

Randomisation was performed using a permuted block randomisation method with decreasing block size. In Year 1, randomisation was stratified by centre. In Year 2, randomisation was stratified by centre and by vaccine actually received by each subject in Year 1. In Year 3, randomisation was again stratified by centre and by vaccine received actually received in Years 1 and 2. This randomisation strategy ensured that, each year, a comparable number of subjects were included in each vaccine group with an equal distribution of subjects vaccinated intradermally or intramuscularly in previous years (Figure 1). An interactive voice response system via telephone allocated a dose number to each subject that corresponded to one of the vaccines. In Year 3, an additional randomisation identified a subset of subjects who would provide blood samples for immunogenicity analysis after the third vaccination. The Year 3 blood sampling randomisation list took into account the route of vaccination used for each subject in each of the 3 years so that the immunogenicity subset contained comparable numbers from each combination of ID and IM vaccination over 3 years (Figure 1). It was initially planned to have a similar immunogenicity subset for Year 2. However, as neither of the ID formulations tested in Year 1 met the non-inferiority criteria, the protocol was amended (and renewed informed consent obtained) to collect blood from all subjects to compare the 9 μg ID vaccine with the 15 μg IM formulation. The study was open-label, except for antigen dosage in the two ID groups in Year 1, which was double-blind.

### Vaccination

Both the investigational ID vaccines and the IM control vaccine (Vaxigrip^®^) were trivalent inactivated, split-virion influenza vaccines (Sanofi Pasteur, Lyon, France). The antigenic dosage and strain composition of the investigational ID vaccines and the IM control vaccine given each year is shown in Table 1. The injected volume was 0.1 ml for all ID vaccines and 0.5 ml for the IM vaccine. ID vaccination was performed using the microinjection system described above. All vaccines were given into the deltoid region.

**Table 1:** Virus strain composition and antigenic content of each annual influenza vaccine

Vaccine dosage and route*	**Strain composition**
Year 1:	Northern Hemisphere 2003–2004 formulation:
3 μg ID or	A/New Caledonia/20/99 (H1N1) (A/New Caledonia/20/99-like strain)
6 μg ID or	A/Panama/2007/99 (H3N2) (A/Moscow/10/99-like strain)
15 μg IM	B/Shandong/7/97 (B/Hong Kong/330/2001-like strain)

Year 2:	Northern Hemisphere 2004–2005 formulation:
9 μg ID or	A/New Caledonia/20/99 (H1N1) (A/New Caledonia/20/99-like strain)
15 μg IM	A/Wyoming/3/2003 (H3N2) (A/Fujian/411/2002-like strain) B/Jiangsu/10/2003 (B/Shanghai/361/2002-like strain)

Year 3:	Southern Hemisphere 2005 formulation:
9 μg ID, or	A/New Caledonia/20/99 IVR-116 (H1N1) (A/New Caledonia/20/99-like strain)
15 μg IM	A/Wellington/1/2004 IVR-139 (H3N2) (A/Wellington/1/2004-like strain) B/Jiangsu/10/2003 (B/Shanghai/361/2002-like strain)

*Intradermal (ID) vaccines were formulated to contain 3, 6, 9 μg of haemagglutinin (HA) per strain in a volume of 0.1 ml and administered by microinjection; the intramuscular (IM) vaccine was the licensed vaccine Vaxigrip^®^, containing 15 μg of (HA) per strain in a volume of 0.5 ml.

### Immunogenicity outcomes

The primary endpoint was the geometric mean titre (GMT) of anti-HA antibodies for each of the three influenza strains 21 days after the first vaccination in each group. Antibody titres were determined in duplicate simultaneously for each strain before and 21 days after vaccination by the HA inhibition assay using the strains included in the vaccine each year [24] and are presented as the highest reciprocal dilution which induced complete HA inhibition. Strain-specific GMTs were also determined before and 21 days after the second and third vaccinations in each group (Table 1). Immunogenicity was further assessed by calculating the following for each strain in each group: geometric mean ratio of post-vaccination titre to pre-vaccination titre (GMTR); seroprotection rate (percentage of subjects with a post-vaccination titre ≥ 40); and seroconversion or significant titre increase rate (post-vaccination titre ≥ 40 in subjects with a pre-vaccination titre <10 or a ≥ 4-fold increase in titre after vaccination in subjects with a pre-vaccination titre ≥ 10). Compliance with the immunogenicity criteria for people aged 18 to 60 years outlined in the EMEA Note for Guidance was determined. The EMEA recommendations are that at least one of the following criteria should be met for each strain: GMTR ≥ 2.5, seroprotection rate >70%, and seroconversion or significant increase rate >40% [23].

### Safety outcomes

The following solicited reactions based on the EMEA Note for Guidance [23], occurring within 3 days of each vaccination were recorded: injection site induration >5 cm for more than 3 days, injection site bruising, fever (axillary body temperature increased by >37.5°C and lasting ≥ 24 hours), malaise and shivering. Additionally, subjects recorded the occurrence of any solicited injection site reactions (pain, pruritus and any of the following of at least 0.5 cm in diameter: redness, induration, oedema and bruising) and systemic reactions (axillary temperature >37.5°C, asthenia, headache, arthralgia, myalgia, rigors, sweating and malaise) within 7 days of vaccination. Safety results are also presented according to vaccination history during the study. Details of any serious adverse events (SAEs) were collected up to 6 months after the last vaccination.

### Statistical methods

Assuming a standard deviation of 0.67 for the difference in log-transformed GMTs, it was calculated that 344 subjects per group were necessary to show non-inferiority (that is, a minimum acceptable post-vaccination GMT ratio [ID/IM] of 1/1.5) with a global power of 80.4% (93% for the individual test for each strain) and a significance level of 5%. To allow for 10% of subjects not being evaluable, 1146 subjects were to be included, 382 in each group.

All statistical analyses were performed using SAS software, version 8.2 (SAS Institute, Cary, NC, USA). The primary endpoint was analysed to determine whether the ID vaccine was non-inferior to IM vaccination. For both ID vaccine doses used in the first year, the ratio of GMTs after the first vaccination (ID/IM) and their 95% confidence intervals (CI) were calculated for each strain. To conclude non-inferiority of the ID vaccine, the lower bound of the 95% CI had to be greater than 1/1.5 for each strain. This analysis was performed, as planned in the protocol, on data collected from all study participants after the first vaccination and was performed with the aim of selecting one of the two ID vaccine formulations for further analysis in Years 2 and 3. If both the 3 μg and 6 μg ID vaccines had been found to be non-inferior to the IM control, then the lower of the two would have been selected. The ratio of GMTs (ID/IM) and their 95% CIs were also calculated to compare the immunogenicity of the ID 9 μg vaccine with the IM 15 μg vaccine after the second vaccination. 95% CIs were calculated for all immunogenicity outcomes using the normal approximate method for GMTs and GMTRs and using the Clopper-Pearson method for single percentages [25]. Safety outcomes are presented descriptively.

The primary analysis in Year 1 included all subjects who had conformed with the protocol and according to vaccine group allocation (per protocol analysis). Immunogenicity analysis after each vaccination was carried out on all subjects who had received all vaccinations up to that point and for whom pre- and post-vaccination titres were available. Safety analysis was performed on all available data.

## Results

### Subjects

**Figure 2:**
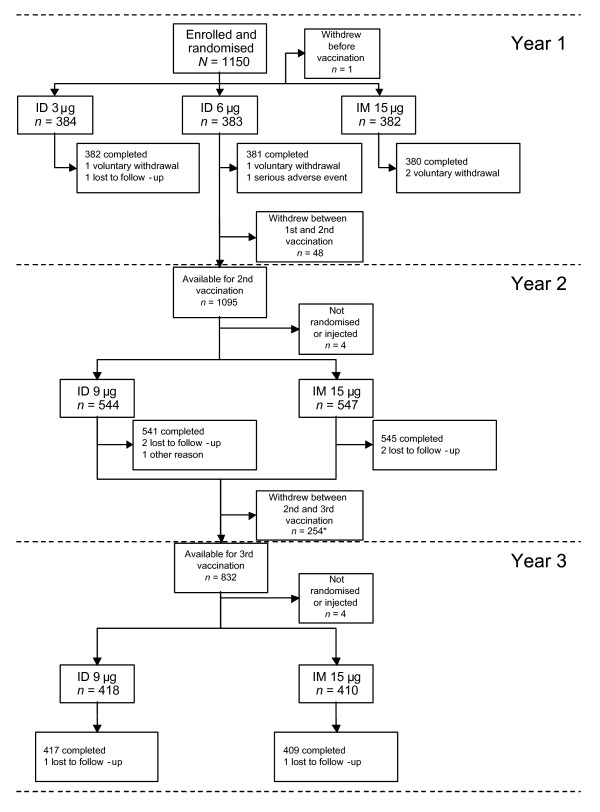
**Study flow chart**. ID, intradermal; IM, intramuscular; *N*, number of subjects. *As one of the study centres (the Czech centre) did not participate in the third part of the study, a large number of subjects were discontinued from the study between the second and third vaccinations.

Of the 1150 subjects enrolled and randomised, 1149 were vaccinated in Year 1, 1091 were re-randomised and vaccinated in Year 2, and 828 were re-randomised and vaccinated in Year 3 (Figure 2).

One subject was withdrawn before completing the first part of the study due to an SAE unrelated to vaccination (hospitalisation for a chronic psychiatric condition). Four subjects died during the study; cause of death hepatocellular carcinoma, myosarcoma of the left thigh adductor, cerebral haemorrhage and myocardial infarction (98 other subjects experienced an SAE during the 3 years of the study). None were related to vaccination.

Groups were equally matched in terms of both age and sex ratio; more women than men were enrolled (Table 2).

**Table 2:** Demographic and baseline characteristics

	**Year 1**			
	**3 μg ID** **(*N *= 378)**	**6 μg ID** **(*N *= 375)**	**15 μg IM** **(*N *= 376)**	**Total** **(*N *= 1129)**
	
Mean age ± standard deviation	39 ± 11	39 ± 12	39 ± 12	39 ± 12
Male/female ratio	0.6	0.7	1.0	0.7
History of influenza vaccination*, *n *(%)	112 (29.6)	114 (30.4)	100 (26.6)	326 (28.9)
Reaction to previous influenza vaccination*, *n *(%)	10 (8.9)	9 (7.9)	7 (7.0)	26 (8.0)

	**Year 2**			

	**9 μg ID** **(*N *= 544)**		**15 μg IM** **(*N *= 547)**	**Total** **(*N *= 1091)**
	
mean age +/- standard deviation	40 ± 12		40 ± 12	40 ± 11
Male/female ratio	0.7		0.7	0.7

	**Year 3**			

	**9 μg ID** **(*N *= 417)**		**15 μg IM** **(*N *= 411)**	**Total** **(*N *= 828)**
	
mean age +/- standard deviation	41 ± 11		40 ± 11	40 ± 11
Male/female ratio	0.8		0.7	0.7

Data are for the per protocol population for Year 1 and the safety population for Years 2 and 3. ID, intradermal; IM, intramuscular; *N *and *n*, number of subjects

*Subjects who recalled having previously received an influenza vaccination and having experienced any reactions after a previous influenza vaccination.

### Immunogenicity

The 6 μg ID vaccination induced an immune response that satisfied the EMEA immunogenicity criteria (all criteria met for all strains, except the seroprotection and seroconversion rates for the B strain) (Table 3). However, neither the 3 μg ID nor the 6 μg ID formulations met the pre-defined non-inferiority criteria. The lower boundary of the 95% CI for the ratio of post-vaccination GMTs (ID/IM) was lower than 1/1.5 in both ID vaccine groups for each strain (data not shown). Post-vaccination GMTs were lower in the ID vaccine groups than in the IM vaccine group for all three strains.

**Table 3:** Comparison of immunogenicity results after intradermal or intramuscular vaccination

	European Medicines Evaluation Agency criteria	**A/H1N1**			**A/H3N2**			**B**		
**Vaccine received in Year 1**		**3 μg ID**	**6 μg ID**	**15 μg IM**	**3 μg ID**	**6 μg ID**	**15 μg IM**	**3 μg ID**	**6 μg ID**	**15 μg IM**

Geometric mean titre (95% CI)	-	93.6 (78.5;112)	110 (90.5;133)	206 (177;239)	132 (117;149)	156 (137;178)	300 (266;337)	19.2 (17.1;21.5)	21.7 (19.3;24.5)	40.8 (36.1;46.2)
Seroprotection rate*, % (95% CI)	>70	72.7 (67.9;77.1)	71.3 (66.5;75.8)	87.1 (83.3;90.3)	88.5 (84.9;91.5)	88.2 (84.5;91.3)	96.9 (94.6;98.4)	28.5 (23.9;33.3)	32.9 (28.1;37.9)	55.7 (50.5;60.8)
Seroconversion^†^/significant increase rate^‡^, % (95% CI)	>40	53.1 (48.0;58.2)	55.1 (50.0;60.1)	75.0 (70.3;79.3)	35.4 (30.6;40.4)	43.0 (38.0;48.2)	63.8 (58.7;68.6)	21.0 (17.0;25.5)	27.3 (22.8;32.1)	47.7 (42.6;52.9)
Geometric mean titre ratio (post-/pre-vaccination) (95% CI)	2.5	7.32 (6.16;8.7)	8.38 (6.94;10.1)	17.4 (14.7;20.5)	3.48 (3.02;4.01)	4.19 (3.58;4.90)	9.11 (7.71;10.8)	2.38 (2.13;2.67)	2.73 (2.42;3.07)	4.97 (4.37;5.67)

**Vaccine received in Year 2**		**9 μg ID**		**15 μg IM**	**9 μg ID**		**15 μg IM**	**9 μg ID**		**15 μg IM**

Geometric mean titre (95% CI)	-	180 (161; 202)		192 (174; 212)	380 (343; 420)		386 (355; 420)	80.6 (71.6; 90.7)		83.1 (74.8; 92.3)
Seroprotection rate*, % (95% CI)	>70	90.0 (87.1; 92.4)		93.4 (90.0; 95.3)	97.2 (95.4; 98.4)		99.4 (98.4; 99.9)	73.0 (69.1; 76.8)		74.4 (70.5; 78.0)
Seroconversion^†^/significant increase rate^‡^, % (95% CI)	>40	43.0 (38.8; 47.3)		45.7 (41.4; 50.0)	53.1 (48.8; 57.4)		50.8 (46.5; 55.1)	63.4 (59.2; 67.5)		66.6 (62.5; 70.6)
Geometric mean titre ratio (post-/pre-vaccination) (95% CI)	2.5	4.3 (3.8; 4.8)		4.7 (4.2; 5.3)	4.4 (4.0; 5.0)		4.4 (3.9; 5.0)	7.8 (7.0; 8.8)		8.3 (7.5; 9.1)

**Vaccine received in Year 3**		**9 μgID**		**15 μgIM**	**9 μgID**		**15 μgIM**	**9 μgID**		**15 μgIM**

Geometric mean titre (95% CI)	-	127 (103; 158)		117.8 (92.7; 149.6)	415 (337; 510)		300 (242; 373)	91.0 (74.5; 111.1)		86.2 (68.4; 108.7)
Seroprotection rate*, % (95% CI)	>70	90.7 (83.6; 95.5)		91.3 (84.2; 96.0)	100 (96.6; 100)		99.0 (94.8; 100.0)	83.3 (74.9; 89.8)		81.4 (72.4; 88.4)
Seroconversion^†^/significant increase rate^‡^, % (95% CI)	>40	14.8 (8.7; 22.9)		18.3 (11.4; 27.1)	60.2 (50.3; 69.5)		45.2 (35.4; 55.3)	24.1 (16.4; 33.3)		19.6 (12.4; 28.6)
Geometric mean titre ratio (post-/pre-vaccination) (95% CI)	2.5	2.0 (1.7; 2.4)		2.1 (1.7; 2.5)	4.6 (3.8; 5.6)		3.5 (2.8; 4.4)	2.3 (1.9; 2.7)		2.3 (1.9; 2.8)

CI, confidence interval; ID, intradermal; IM, intramuscular.

*Defined as antibody titre ≥ 40; ^†^for subjects with a titre <10 on day 0, defined as a post-injection titre ≥ 40; ^‡^for subjects with a titre ≥ 10 on day 0, defined as a ≥ 4-fold increase in titres. Data are from the other immunogenicity (OI) population.

After the second vaccination in Year 2, the immune responses against all strains were comparable with the 9 μg ID and control vaccine and all EMEA criteria were met for all strains in both groups (Table 3). For each strain, the GMT ratio between the ID and IM groups was close to 1 with a narrow 95% CI (A/H1N1: 0.94 [0.81; 1.09], A/H3N2: 0.98 [0.86; 1.12], B: 0.97 [0.83; 1.14]). Similarly, comparable results between the two groups were obtained after the Year 3 vaccination for the subset assessed. In both groups, all three EMEA criteria were met for the A/H3N2 strain, and the seroprotection criterion was met for the A/H1N1 and B strains.

### Safety

After vaccination in Years 1, 2 and 3, the incidence of EMEA-specified reactions, most commonly shivering and malaise, was comparable between groups (Table 4). Considering the incidence of these reactions after Years 2 and 3 vaccinations in relation to the vaccination route in Years 1 and 2, prior vaccination route was seen to have no apparent effect on reactogenicity in subsequent years. In Year 3, the incidence of EMEA reactions did not differ substantially between all sub-groups, that is, it did not depend on whether previous vaccinations had been via ID or IM routes. Notably, one or two previous ID vaccinations did not increase the reactogenicity to ID vaccination in Year 3 (in comparison with those subjects who had been vaccinated with the IM control vaccine in the first 2 years). Nor did prior ID vaccination increase the reactogenicity of the IM vaccine in Year 2 or 3. Similar results were obtained when considering the incidence of solicited injection site and systemic reactions within 7 days of vaccination in each subgroup (data not shown).

**Table 4:** Safety summary: number of subjects with European Medicines Evaluation Agency -specified reactions after each intradermal and intramuscular vaccination according to vaccine history during the study

	**Year 1**									
	**3 μg ID (*N *= 384)**				**6 μg ID (*N *= 383)**			**15 μg IM (*N *= 382)**		
	
≥ 1 EMEA reaction, *n *(%)	38 (9.9)				39 (10.2)			50 (13.1)		
Injection site induration*	0				0			1 (0.3)		
Injection site ecchymosis (bruising)	2 (0.5)				1 (0.3)			7 (1.8)		
Fever^†^	4 (1.0)				5 (1.3)			6 (1.6)		
Malaise	12 (3.1)				17 (4.4)			19 (5.0)		
Shivering (rigors)	26 (6.8)				26 (6.8)			21 (5.5)		

	**Year 2 (vaccine history and group)**									

	ID/ID (*N *= 363)		IM/ID (*N *= 181)		**ID total (*N *= 544)**		ID/IM (*N *= 363)	IM/IM (*N *= 184)		**IM total (*N *= 547)**
	
≥ 1 EMEA reaction, *n *(%)	41 (11.3)		20 (11.0)		**61 (11.2)**		39 (10.7)	28 (15.2)		**67 (12.2)**
Injection site induration*	0		0		**0**		0	0		**0**
Injection site ecchymosis (bruising)	5 (1.4)		2 (1.1)		**7 (1.3)**		7 (1.9)	3 (1.6)		**10 (1.8)**
Fever^†^	3 (0.8)		2 (1.1)		**5 (0.9)**		3 (0.8)	0		**3 (0.5)**
Malaise	19 (5.2)		8 (4.4)		**27 (5.0)**		17 (4.7)	15 (8.2)		**32 (5.9)**
Shivering (rigors)	27 (7.4)		9 (5.0)		**36 (6.6)**		23 (6.3)	18 (9.8)		**41 (7.5)**

	**Year 3 (vaccine history and group)**									

	ID/ID/ID (*N *= 137)	IM/ID/ID (*N *= 71)	ID/IM/ID (*N *= 138)	IM/IM/ID (*N *= 72)	**ID total (*N *= 418)**	ID/ID/IM (*N *= 132)	IM/ID/IM (*N *= 72)	ID/IM/IM (*N *= 134)	IM/IM/IM (*N *= 72)	**IM total (*N *= 410)**
	
≥ 1 EMEA reaction, *n *(%)	13 (9.5)	11 (15.5)	19 (13.8)	11 (15.3)	**54 (12.9)**	18 (13.6)	6 (8.3)	13 (9.7)	10 (13.9)	**47 (11.5)**
Injection site induration*	0	0	0	0	**0**	0	0	0	0	**0**
Injection site ecchymosis (bruising)	4 (2.9)	3 (4.2)	2 (1.4)	4 (5.6)	**13 (3.1)**	3 (2.3)	1 (1.4)	3 (2.2)	4 (5.6)	**11 (2.7)**
Fever^†^	3 (2.2)	1 (1.4)	3 (2.2)	0	**7 (1.7)**	1 (0.8)	0	0	1 (1.4)	**2 (0.5)**
Malaise	3 (2.2)	5 (7.0)	6 (4.3)	2 (2.8)	**16 (3.8)**	9 (6.8)	1 (1.4)	6 (4.5)	4 (5.6)	**20 (4.9)**
Shivering (rigors)	8 (5.8)	4 (5.6)	12 (8.7)	7 (9.7)	**31 (7.4)**	11 (8.3)	4 (5.6)	6 (4.5)	5 (6.9)	**26 (6.3)**

Data are from the safety population. EMEA, European Medicines Evaluation Agency; ID, intradermal; IM, intramuscular; *n*, number of subjects reporting the event; *N*, number of subjects in the subgroup.

*>5 cm for more than 3 days; ^†^axillary temperature >37.5°C for ≥ 24 hours.

### Solicited reactions within 7 days of vaccination in Years 2 and 3

Injection site pain and bruising occurred at a comparable rate in each group after vaccination with 9 μg ID or the control vaccine in Years 2 and 3 (Table 5). Other solicited injection site reactions, particularly erythema, were more frequent with 9 μg ID than with the IM control. Erythema was the most common local reaction with ID vaccination, typically appearing within 3 days of vaccination and resolving spontaneously within 7 days. Injection site reactions were mostly mild or moderate: 90% of all injection site reactions observed with 9 μg ID and 96% with the IM control were mild to moderate. In particular, after each vaccination no more than six subjects (1.4%) per group reported severe pain, defined as 'pain preventing normal daily activity', or pruritus, defined as 'continuous pruritus' (other injection site reactions were considered severe if they measured more than 5 cm in diameter). The 9 μg ID and the control vaccine caused comparable numbers of solicited systemic reactions, the most frequent of which were asthenia and headache (Table 5). Grade 3 or 'severe' (defined as 'preventing normal daily activity') systemic reactions concerned no more than 4% of subjects vaccinated intradermally and 2.7% or less of subjects vaccinated intramuscularly. These reactions typically occurred within 3 days of vaccination and spontaneously resolved within 3 days.

**Table 5:** Summary of solicited injection site and systemic reactions within 7 days of intradermal or intramuscular influenza vaccination

	**Year 2**		**Year 3**	
	**9 μgID** **(*N *= 544)**	**15 μg IM** **(*N *= 547)**	**9 μg ID** **(*N *= 418)**	**15 μg IM (*N *= 410)**

Solicited injection site reactions, *n *(%)	420 (77.2)	253 (46.3)	317 (75.8)	186 (45.5)
Erythema				
>0.5 cm	378 (69.5)	53 (9.7)	274 (65.6)	50 (12.2)
>5 cm	29 (5.4)	3 (0.6)	25 (6.0)	2 (0.5)
Induration				
>0.5 cm	212 (39.0)	47 (8.6)	166 (39.7)	49 (12.0)
>5 cm	7 (1.3)	2 (0.4)	2 (0.5)	2 (0.5)
Oedema				
>0.5 cm	233 (42.8)	28 (5.1)	150 (35.9)	33 (8.0)
>5 cm	9 (1.7)	2 (0.4)	7 (1.7)	2 (0.5)
Bruising				
>0.5 cm	10 (1.8)	10 (1.8)	14 (3.3)	13 (3.2)
>5 cm	0	1 (0.2)	1 (0.2)	2 (0.5)
Pain				
any grade	204 (37.5)	214 (39.1)	180 (43.1)	152 (37.1)
grade 3	3 (0.6)	2 (0.4)	5 (1.2)	4 (1.0)
Pruritus				
any grade	172 (31.6)	39 (7.1)	121 (28.9)	31 (7.6)
grade 3	3 (0.6)	1 (0.2)	6 (1.4)	1 (0.2)

Solicited systemic reactions, *n *(%)	155 (28.5)	181 (33.1)	123 (29.4)	101 (24.6)
Pyrexia				
>37.5°C	8 (1.5)	5 (0.9)	10 (2.4)	2 (0.5)
>38.5°C	0	0	2 (0.5)	1 (0.2)
Asthenia				
any grade	97 (17.8)	108 (19.7)	75 (17.9)	52 (12.7)
grade 3	11 (2.0)	6 (1.1)	8 (1.9)	6 (1.5)
Headache				
any grade	88 (16.2)	82 (15.0)	69 (16.5)	57 (13.9)
grade 3	7 (1.3)	4 (0.7)	3 (0.7)	3 (0.7)
Arthralgia				
any grade	31 (5.7)	33 (6.0)	26 (6.2)	8 (2.0)
grade 3	1 (0.2)	2 (0.4)	2 (0.5)	0
Myalgia				
any grade	40 (7.4)	84 (15.4)	47 (11.2)	41 (10.0)
grade 3	1 (0.2)	4 (0.7)	2 (0.5)	2 (0.5)
Rigors				
any grade	41 (7.5)	44 (8.0)	35 (8.4)	31 (7.6)
grade 3	1 (0.2)	3 (0.6)	3 (0.7)	1 (0.2)
Increased sweating				
any grade	35 (6.4)	39 (7.1)	28 (6.7)	25 (6.1)
grade 3	2 (0.4)	2 (0.4)	0	2 (0.5)
Malaise				
any grade	31 (5.7)	37 (6.8)	18 (4.3)	22 (5.4)
grade 3	3 (0.6)	4 (0.7)	2 (0.5)	1 (0.2)

Data are from the safety population. ID, intradermal; IM, intramuscular; *n*, number of subjects reporting the event; *N*, number of subjects in the subgroup; %, *n*/(*N *minus the number of subjects with missing data for the given variable).

Pain: Grade 1: mild/well tolerated, Grade 2: moderate/hindering movement, Grade 3 pain: preventing normal daily activity. Pruritus: Grade 1: mild/occasional, Grade 2: moderate/frequent, Grade 3 severe/continuous. Asthenia, headache, arthralgia, myalgia, rigors, increased sweating, malaise: Grade 1: symptom present but well tolerated, Grade 2: symptom interfered with normal daily activities, Grade 3 symptom prevented normal daily activities.

## Discussion

The primary aim of this study was to identify the HA dose that, when administered intradermally using a microinjection system, would elicit an immune response that is statistically non-inferior to that elicited by a standard IM vaccination of 15 μg of HA/strain. In line with previous observations [19,20,26], ID vaccination with either 3 μg or 6 μg of HA was immunogenic and, in the case of the 6 μg vaccine, sufficiently immunogenic to comply with the EMEA immunogenicity recommendations [23]. However, antibody responses to 6 μg were lower than with the standard IM vaccine and non-inferiority was not demonstrated. These results are consistent with those of a large study in healthy adults (20 to 50 years), in which a reduced-dose ID influenza vaccination led to inferior antibody responses compared with a standard IM vaccine, despite meeting the EMEA requirements [26]. Two smaller studies have shown comparable immunogenicity of reduced-dose ID influenza to full-dose IM vaccines in adults aged between 18 and either 40 or 60 years [19,20]. In our study, as non-inferiority was not demonstrated with either 3 μg or 6 μg of HA, we amended the protocol to continue vaccination in Years 2 and 3 with an escalated ID dose of 9 μg of HA/strain. This allowed the immunogenicity of this higher dose to be evaluated descriptively in preparation for a follow-up study that would be needed to repeat the formal non-inferiority analysis for such an escalated dose.

Despite the lower antigen content, the ID 9 μg vaccine was comparably immunogenic to the reference IM vaccine, satisfying EMEA criteria for all three virus strains. Although this study did not compare the same dosage given by ID and IM routes, these results are consistent with previous studies with rabies, hepatitis B and influenza vaccines that support the higher immunogenicity of the ID vaccination route [13-17]. In a recently reported study of an investigational ID influenza for elderly adults, an ID vaccination with 15 μg of HA per strain was shown to elicit significantly higher immune responses than the IM vaccination, also with 15 μg HA per strain [27]. The ID vaccine evaluated in elderly adults by Holland et al. [27] was manufactured by Sanofi Pasteur (the manufacturers of the vaccines tested in our study), was administered using the same microinjection system, and was studied in comparison with the same control vaccine (Vaxigrip^®^) as in our study.

Reactogenicity of the ID vaccine was comparable to that of the IM vaccine in terms of both EMEA reactions and solicited systemic reactions. It should be noted that the reactions listed in the EMEA Note for Guidance were specifically designed to determine the reactogenicity of IM vaccines and, as such, may not be fully appropriate for assessing reactogenicity after ID vaccination [23]. As would be expected with a vaccine injected into the skin in comparison with an injection deep into the muscle, recipients of the ID vaccine more frequently reported local reactions at the injection site within 7 days of vaccination, particularly erythema. Importantly, these reactions were not associated with an increased incidence of injection site pain. Other studies have also shown increased local inflammation (mainly erythema and induration) to ID influenza vaccination compared with IM vaccination, but with a similar or lower incidence of injection site pain [19,20,26]. This increase in local reactions is linked to the underlying inflammatory or immunological response in the skin, which is more visible with ID than IM vaccination.

It has been proposed that delivery of antigen via the ID route and associated activation of dermal dendritic cells favours the induction of a Th1-type cellular immune response, which can lead to delayed-type hypersensitivity [28,29]. Repeated ID vaccination may, therefore, increase the risk of delayed-type hypersensitivity local reactions. In a study of a hepatitis B vaccine, secondary systemic or local reactions were more frequent with a mixed vaccine schedule (IM followed by ID or vice versa) than either an IM/IM or an ID/ID schedule [30]. In our study, the safety profile after three ID vaccinations appeared similar to that after a single ID vaccination, suggesting that influenza ID vaccination can be repeated annually without increasing reactogenicity. Furthermore, interchanging the IM and ID vaccines from one year to the next did not adversely affect the safety profile.

Despite the documented health and economic burden of influenza disease in adults younger than 60 years [31-34], surveys show that vaccine uptake is lower than the target coverage rates of between 50% to 90% set by national and international health organisations [35-37]. Coverage rates of as low as 10% have been reported among Western European adults aged 20 to 40 years, with slightly higher rates, 15% to 20%, in adults aged 40 to 60 years [38]. These rates are comparable with those reported in the USA, as well as in countries in Asia, Latin America and Eastern Europe [39,40]. Several studies have investigated why vaccine uptake remains low. In two recent reports from Europe and the USA, 14% to 16% of individuals questioned cited a dislike of needles and injections as one of the reasons (although not the primary reason) for not getting vaccinated [38,41]. This finding suggests that alternative vaccination methods that do not use a classic syringe and needle have the potential to contribute to increase vaccination coverage among such populations. The ID vaccine investigated in our study may represent such an alternative method. This vaccine used a newly developed microinjection system designed as an easy-to-use system with a very narrow, 1.5 mm long needle that is inserted perpendicularly into the skin to accurately inject antigen into the dermis [22]. This ID influenza vaccine thus provides an alternative to IM vaccine for adults younger than 60 years that is convenient for the healthcare provider and may contribute to increase vaccine uptake in this population.

As the primary outcome was not met in the first year of the study, our findings are limited by the fact that the immunogenicity of the 9 μg ID vaccine was descriptively compared with the IM vaccine after the second and third vaccinations. A formal statistical comparison of the 9 μg ID vaccine dose to the IM vaccine was not possible, as the population was not representative of an ID vaccination-naïve population. This formal comparison was done in a second trial that has been reported separately [42]. These trials formed part of a marketing authorisation application, which has been approved by the European Commission and will be marketed in the European Union under the trade names Intanza^® ^and IDflu^®^.

## Conclusion

We have shown that a reduced dose, 9 μg/strain, of trivalent, inactivated, split-virion, seasonal influenza vaccine in a lower injection volume given intradermally using a novel microinjection system is as immunogenic as conventional IM vaccine with a comparable safety profile. Furthermore, our results show that the ID vaccine can be re-administered or interchanged with the IM vaccine annually without adversely affecting the safety profile. This vaccine administered using microinjection presents a promising alternative to IM vaccine for the vaccination of adults younger than 60 years against seasonal influenza.

## Abbreviations

CI: confidence interval; EMEA: European Medicines Evaluation Agency; GMT: geometric mean titre; GMTR: geometric mean titre ratio; HA: haemagglutinin; ID: intradermal; IM: intramuscular; SAE: serious adverse event.

## Competing interests

FW and CS are employees of Sanofi Pasteur, the sponsor of this study. All other authors have no competing interests.

## Authors' contributions

The trial investigators JB, AA, AL, NM, PB, YC, ED, SH and PVB enrolled and followed subjects, revised and approved the final manuscript for publication. FW conceived the study, participated in its design, helped draft the manuscript and approved the final manuscript for publication, and was also the sponsor's responsible medical officer for the study. CS participated in the statistical design of the study, performed the statistical analysis and approved the final manuscript for publication.

## Pre-publication history

The pre-publication history for this paper can be accessed here:


